# Identification of sequestered chloroplasts in photosynthetic and non-photosynthetic sacoglossan sea slugs (Mollusca, Gastropoda)

**DOI:** 10.1186/1742-9994-11-15

**Published:** 2014-02-21

**Authors:** Gregor Christa, Katharina Händeler, Till F Schäberle, Gabriele M König, Heike Wägele

**Affiliations:** 1 Zoologisches Forschungsmuseum Alexander Koenig, Adenauerallee 160, 53113 Bonn, Germany; 2 Institute for Pharmaceutical Biology, University of Bonn, Nussallee 6, 53115 Bonn, Germany

**Keywords:** DNA-barcoding, Food analyses, Kleptoplasty, *rbcL*, Sacoglossa, *tufA*

## Abstract

**Background:**

Sacoglossan sea slugs are well known for their unique ability among metazoans to incorporate functional chloroplasts (kleptoplasty) in digestive glandular cells, enabling the slugs to use these as energy source when starved for weeks and months. However, members assigned to the shelled Oxynoacea and Limapontioidea (often with dorsal processes) are in general not able to keep the incorporated chloroplasts functional. Since obviously no algal genes are present within three (out of six known) species with chloroplast retention of several months, other factors enabling functional kleptoplasty have to be considered. Certainly, the origin of the chloroplasts is important, however, food source of most of the about 300 described species is not known so far. Therefore, a deduction of specific algal food source as a factor to perform functional kleptoplasty was still missing.

**Results:**

We investigated the food sources of 26 sacoglossan species, freshly collected from the field, by applying the chloroplast marker genes *tufA* and *rbcL* and compared our results with literature data of species known for their retention capability. For the majority of the investigated species, especially for the genus *Thuridilla*, we were able to identify food sources for the first time. Furthermore, published data based on feeding observations were confirmed and enlarged by the molecular methods. We also found that certain chloroplasts are most likely essential for establishing functional kleptoplasty.

**Conclusions:**

Applying DNA-Barcoding appeared to be very efficient and allowed a detailed insight into sacoglossan food sources. We favor *rbcL* for future analyses, but *tufA* might be used additionally in ambiguous cases. We narrowed down the algal species that seem to be essential for long-term-functional photosynthesis: *Halimeda, Caulerpa, Penicillus, Avrainvillea, Acetabularia* and *Vaucheria.* None of these were found in *Thuridilla*, the only plakobranchoidean genus without long-term retention forms. The chloroplast type, however, does not solely determine functional kleptoplasty; members of no-retention genera, such as *Cylindrobulla* or *Volvatella,* feed on the same algae as e.g., the long-term-retention forms *Plakobranchus ocellatus* or *Elysia crispata*, respectively. Evolutionary benefits of functional kleptoplasty are still questionable, since a polyphagous life style would render slugs more independent of specific food sources and their abundance.

## Background

Sacoglossa sea slugs are a relatively small group of heterobranch gastropods with currently about 300 species described [1], comprising the shelled Oxynoacea and the shell-less Plakobranchacea. The latter is further divided into the ceras-bearing Limapontioidea (although not monophyletic in phylogenetic analyses) and the parapodia bearing Plakobranchoidea [2-4]. Because of their ability to incorporate functional chloroplasts and subsequently use them for sustenance during starvation over weeks and months Sacoglossa fascinated scientists over decades. This feature is unique among metazoan life forms [3,5-8] and only known elsewhere in members of the Foraminifera (Rhizaria) [9], Ciliophora (Alveolata) [10] and in Dinoflagellata (Alveolata) [11]. Within Sacoglossa usually three states of kleptoplasty are differentiated, either based on measurements of functional chloroplasts with Pulse Amplitude Modulated (PAM) Fluorometry or on CO_2_ fixation experiments [3,12,13]. Following the classification of Händeler et al. [3], specimens that are not able to incorporate chloroplasts functional are considered as no-retention forms (NR); specimens that are able to incorporate functional chloroplasts for up to two weeks of starvation are called short-term-retention forms (StR) and those incorporating functional chloroplasts for over 20 days during starvation are called long-term-retention forms (LtR). The fact that chloroplasts survive for weeks and months in the slug’s digestive system and perform functional photosynthesis, despite the absence of the algal nucleus, has led to the intriguing hypothesis that a horizontal gene transfer must have occurred from the algal organisms into the metazoan life form [7,12,14]. Up to now, a few single genes amplified from *Elysia chlorotica*, Gould [15] were interpreted to encode proteins relevant for photosynthesis, such as *Lhc* (light harvesting complex), *fcp* (fucoxanthin protein), *psbO* (manganese stabilizing protein), and others [7,12,16,17].

Analyzing transcriptomic data for the first time, Wägele et al. [18] found no evidence for a horizontal gene transfer in two sacoglossan species known to maintain chloroplasts for several months, *Elysia timida* Risso [19] and *Plakobranchus ocellatus* van Hasselt [20]. Similar negative results were obtained later for *Elysia chlorotica* by transcriptomic and genomic data [21,22]. Conflicting results of Pierce et al. [23] that suggested a gene transfer based on few single reads in their transcriptomic data, have most recently been interpreted in a different way [24]. Thus, in our view the hypothesis that slugs are able to actively support the kleptoplasts via the translational products of transferred algal genes is rejected and the focus for understanding long-term incorporation of kleptoplasts has to include other factors, like properties of chloroplasts and their origin with regard to functional photosynthesis. We clearly demonstrated this recently by investigating *Plakobranchus ocellatus* during various starvation periods [25]: Despite a broad documented food range in fresh caught animals, only chloroplasts from the ulvophycean *Halimeda macroloba* Decaisne [26] remained in the digestive gland after a starvation period of two months. Unfortunately, our knowledge on sacoglossan food preferences is still scarce. In general the slugs are considered to be stenophagous and mainly sequester members of the Ulvophyceae sensu Floyd & O’Kelly [22,27-33]. Few species (*Elysia crispata, E. clarki and P. ocellatus*) are known to feed on a high variety of algae [18,25,30,34-37]. Only some species are recorded to feed on specific members of Rhodophyta (e.g., *Hermaea bifida*) [38,39], Heterokontophyta (e.g., *Elysia chlorotica*) or sea grasses (*Elysia serca*, Marcus [40]) [3,30,31,41]. Food sources were usually identified by observation, feeding experiments [28,33,42], or by electron microscopically studies of chloroplast types [43,44]. But it is obvious that, especially in potential polyphagous sea slugs, not every food alga may be detected by feeding experiments, in particular when not knowing, which ones to offer. On the other hand, slugs may feed on alternative food sources during food limitation rather than having these as host algae. Molecular barcoding has proved to be a high efficient method to identify algal food sources even when using a single barcoding-marker [30,34-37], instead of two [25]. This now well-established method opens the opportunity to study sacoglossan food sources and origin of kleptoplasts in a highly reliable mode. To find a pattern in chloroplast origin and functional kleptoplasty, a profound database on sequestered chloroplasts for all sacoglossan groups, and especially those taxa hardly studied at all, e.g., the genus *Thuridilla*[18,30], is needed. Following the methods introduced previously [30,34,35] we investigated chloroplast origin in 26 non-starved sacoglossan species, including NR, StR and LtR forms, by DNA-barcoding using the chloroplast markers *tufA* and *rbcL* to enlarge our insight in, and state more precisely, sacoglossan food spectrum. We combined the identified food sources with literature data of species for which retention ability is documented and analyzed in combination with this information if there is a correlation between food sources and retention-form. DNA-barcoding has become an important method in identifying plastid origin in Sacoglossa, but due to varying results and minor pitfalls in the application of *rbcL*, we compared reliability of this gene.

## Results

### Barcoding of *tufA* and *rbcL*

We successfully identified food sources for 30 sacoglossan specimens (overall 26 species) by at least applying one barcoding marker (Additional file 1). For 19 of the 26 species included here we were able to identify the food sources for the first time. For the remaining seven species, we could confirm literature data or even enlarged the food range (Additional file 1). Yet, no *rbcL* amplification products were obtained for four specimens and no *tufA* product was obtained for seven specimens (Additional file 1). *TufA* sequences obtained from *Elysia amakusana* (703) Baba [45], *Elysia* sp. (841), *Cyerce nigra* (860) Bergh [46] and *Costasiella* sp. (863) exclusively represented sequences of bacterial origin.

**Figure 1:**
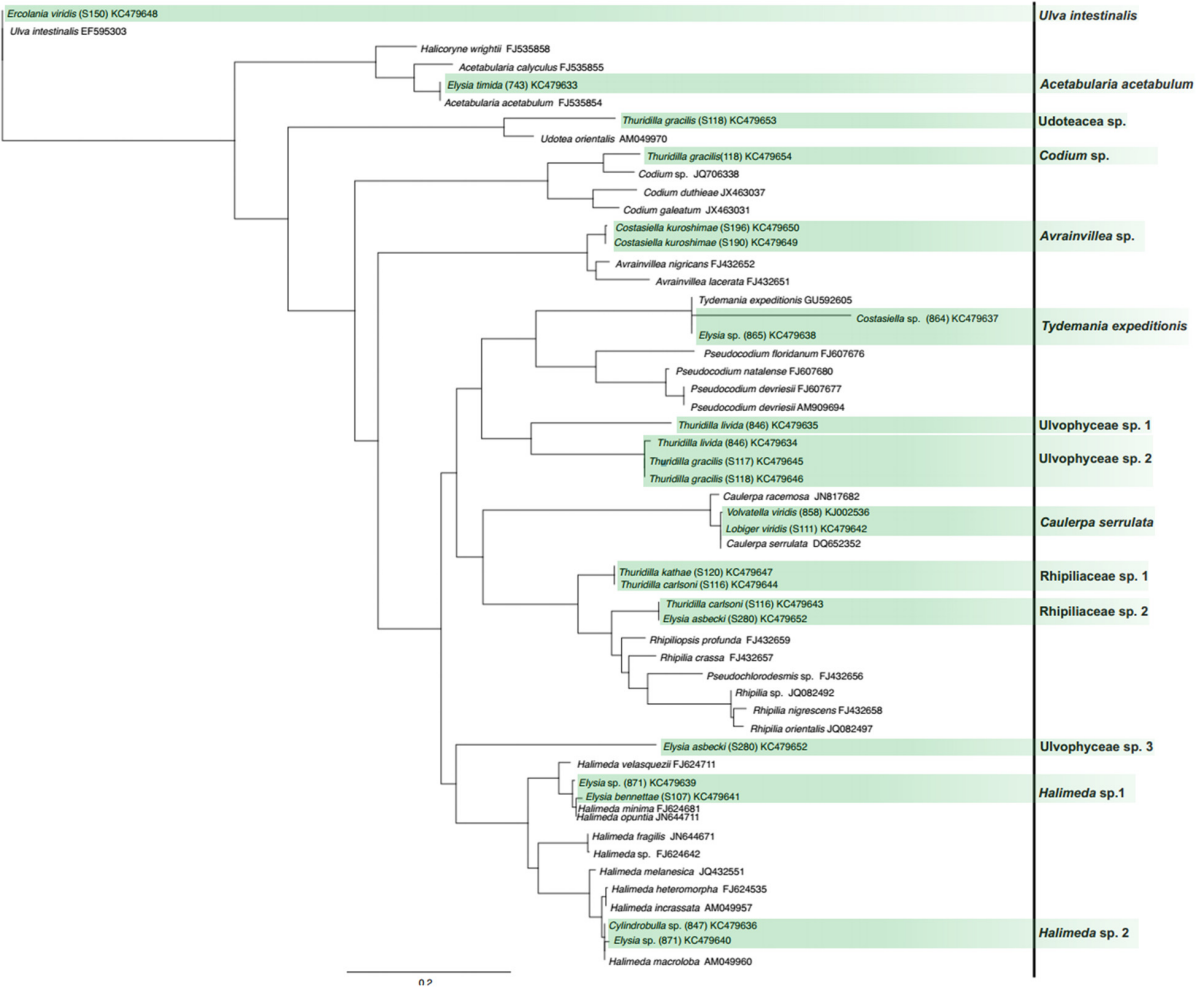
**Food sources identified using *tufA as barcode marker*.** ML tree of identified algal haplotypes in Sacoglossa by using *tufA* (highlighted in green). Identified haplotype is noted on the right side. When sequence match was < 99%, higher taxon name of the algae that formed a monophyletic group with the corresponding haplotype was used. Haplotypes with no monophyletic grouping are named “Ulvophyceae sp.”.

**Figure 2:**
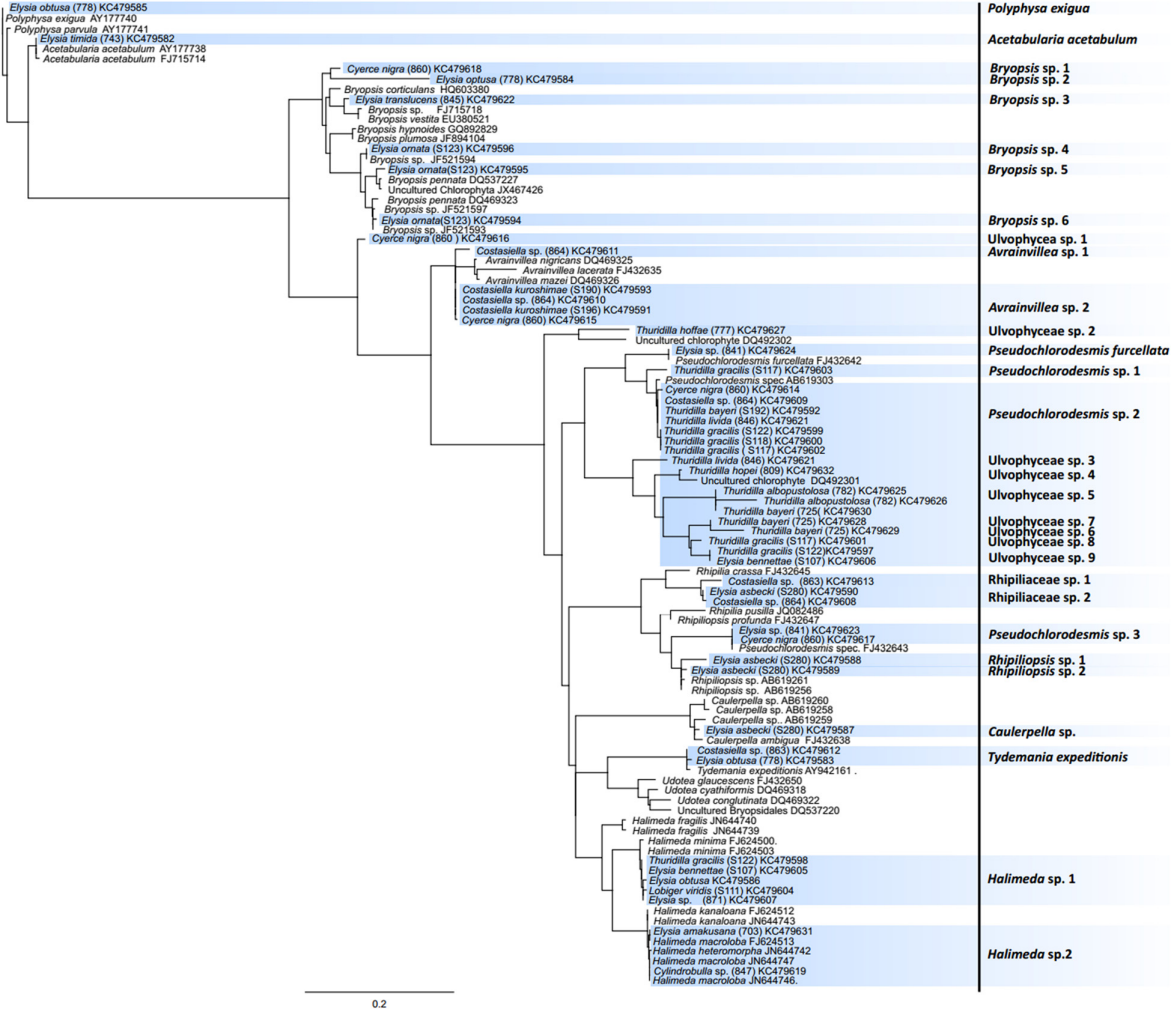
**Food sources identified using *rbcL as barcode marker*.** ML tree of identified algal haplotypes in Sacoglossa by using *rbcL* (highlighted in blue). Identified haplotype is noted on the right side. When sequence match was < 99%, higher taxon name of the algae that formed a monophyletic group with the corresponding haplotype was used. Haplotypes with no monophyletic grouping are named “Ulvophyceae sp.”.

We were able to identify 14 different algal genera combining both markers: eleven genera can be assigned to Bryopsidophyceae, two to Dasycladophyceae and one to Ulvophyceae. *RbcL* revealed about two times more different haplotypes than *tufA* (30 for *rbcL* and 14 for *tufA*, respectively, Figures 1 and 2), though the number of genera is equal (9 for *rbcL* and 10 for *tufA*, respectively). Nine haplotypes for *rbcL* and three haplotypes for *tufA* could not be assigned to a certain algal genus, because of missing reference sequences in GenBank. We are not able to clarify if some of the unidentified haplotypes of *rbcL* and *tufA* actually represent the same algal species. Especially for *rbcL* almost always a higher number of algal haplotypes for a distinct sacoglossan species was revealed, with the highest number of haplotypes for an unknown sister clade of *Pseudochlorodesmis* (7) and for *Bryopsis* (6) (Additional file 1, Figures 1 and 2).

### Food sources and retention form

**Figure 3:**
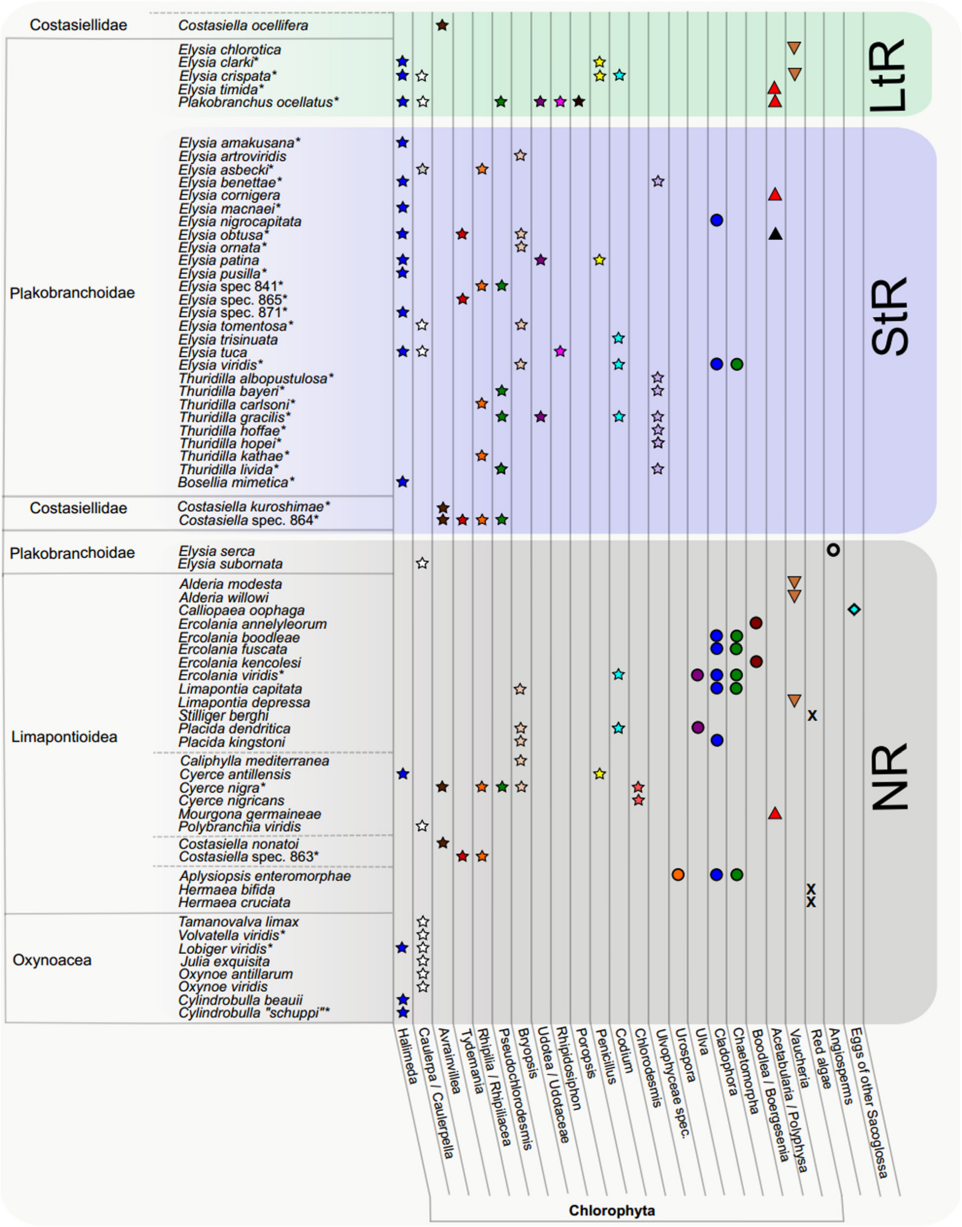
**Food spectrum of Sacoglossa compared to functional retention form.** Food sources of 68 species of Sacoglossa are shown. The classification of Sacoglossa is based on works of [3,4,46]. Asterisk indicates molecular identification of food (overall 33 species). Food sources were either obtained in this study or taken out of literature [3,4,18,25,30,34-37,80-83]. Classification of retention ability was done according to literature data [3,18,47,49,80-84]. **LtR** = Long-term-retention, **StR** = short-term-retention, **NR** = no retention. Stars indicate members of the Bryopsidales, filled circles members of the Ulvophyceae other than Bryopsidales; triangles represent Dasycladales, inverted triangles Heterokontophyta, X red algae, circle sea grass and diamond eggs of other Sacoglossa.

We combined the information obtained from barcoding and literature data, and analyzed this information with regard to the according retention-form. We found that the food of NR forms covers a broad spectrum of ulvophycean algae and includes items that are not consumed by StR or LtR forms: *Boodlea*, *Chlorodesmis*, *Ulva*, *Urospora*, red algae, angiosperms and eggs of other sacoglossans (Figure 3). In contrast, algal taxa like *Poropsis*, *Rhipidosiphon*, *Rhipocephalus*, *Udotea* and an unidentified member of the Ulvophyceae were not identified so far as food sources for NR-forms.

LtR forms consume various ulvophycean algal species, but prefer *Halimeda, Caulerpa, Penicillus* and *Avrainvillea*, the dasycladalen *Acetabularia* and the heterokontophyte *Vaucheria* (Figure 3)*.* However, some NR and LtR forms belonging to different sacoglossan families [5] sequester the same algal species (e.g. *Halimeda*, *Caulerpa*, *Avrainvillea*, *Vaucheria*); therefore a correlation between food sources and functional kleptoplasty cannot be solely algal based. Interestingly, *Thuridilla* species that only comprise StR forms rarely feed on plastids sequestered by LtR forms of *Elysia* and *Plakobranchus ocellatus* (Figure 3)*.*

Within sacoglossan clades, differences in preferred food items exist. All investigated members of the Oxynoacea are NR forms and stenophagous. This is in contrast to all other (non oxynoacean) sacoglossan genera that include polyphagous species (Figure 3). *Cylindrobulla* is the only member of the Oxynoacea that exclusively feeds on *Halimeda,* whereas the members of the remaining Oxynoacea specialized on *Caulerpa* species, with one exception that the *rbcL* analysis in *Lobiger viridis* revealed additional *Halimeda* as food source (Additional file 1, Figure 3). Within Limapontioidea, several species have a broad food spectrum, like *Costasiella* sp. (864) with at least four and *Cyerce nigra* Bergh [46] with at least five different food sources, respectively. However, *Halimeda* and *Caulerpa*, the major oxynoacean food, is scarcely represented amongst the food items of Limapontioidea and only found in *Cyerce antillensis* and *Polybranchia viridis*, respectively. Interestingly, *Costasiella ocellifera* Simroth [48]*,* the only non-plakobranchoid LtR form ([49], unpublished data), feeds exclusively on *Avrainvillea nigricans*.

Four genera of the Plakobranchoidea that all include functional retention forms, are investigated. *Bosellia mimetica* is stenophagous, feeding on *Halimeda*, contrary to the polyphagous *Plakobranchus ocellatus* that feeds on seven different algae (including *Halimeda*, *Caulerpa* and *Udotea)* exhibiting the most diverse food spectrum of all investigated sacoglossans so far (Figure 3); however, these data are obtained from different specimens of different localities [25,37]. The geographically wide distributed taxon *Plakobranchus* might show geographic differences in the consumed food items. Furthermore, cryptic speciation with already ecological differences in feeding can also not be ruled out [50]. Based on the molecular phylogeny of Händeler et al. [3], *Thuridilla* is the sister taxon to *Plakobranchus*, its members principally feeding on a variety of food sources, though *Thuridilla* species neither consume *Halimeda*, nor *Caulerpa* or *Poropsis* (food sources of *Plakobranchus*), but a bryopsidophycean genus related to *Pseudochlorodesmis* (Figure 2). This peculiar food item could not be identified more specifically due to lack of reference sequences in GenBank. Members of the fourth investigated genus, *Elysia,* consume food items recorded also for the shelled sacoglossans, *Halimeda* and *Caulerpa.* Additionally, many *Elysia* species feed on the same bryopsidophycean genera similar to those observed in *Thuridilla* (exceptions see above) or *Plakobranchus*. Some species became very specialized. *Elysia chlorotica* feeds on *Vaucheria* (like the limapontioidean genus *Alderia*), whereas its sister taxon, *Elysia serca*, is reported to feed solely on higher plants (i.e., sea grasses [31]), a unique feature within Sacoglossa. Several species are confirmed in their specific narrow food spectrum, e.g., *Elysia timida*, which exclusively feeds on the dasycladophycean *Acetabularia acetabulum* P.C. Silva [51].

## Discussion

### Food sources and retention form

We analyzed a wide spectrum of sacoglossan sea slugs with regard to their food preferences and plastid origin by using a combination of two barcoding markers, *rbcL* and *tufA*, instead of one [3,18,34-37]. This method proved to be more rapid and more precise in identification of food sources of a large set of Sacoglossa sea slugs, compared to former feeding experiments or feeding observations. Although not every algal food source is identified on species level, the information we provide here increases our knowledge considerably and can now be used for further ecological or behavioral studies.

We found that the ability to perform photosynthesis for at least several days and weeks is established with certain food sources (members of the genera *Elysia, Bosellia* and *Plakobranchus*, see Figure 3 and [3]). At least one of the following six algae, the bryopsidophycean *Halimeda, Caulerpa, Penicillus* and *Avrainvillea,* the heterokontophyte *Vaucheria*, or the dasycladophycean *Acetabularia,* seem to be essential for establishing long-term-retention. However, non-retention forms also feed on these specific algae and are not able to perform photosynthesis. Both aspects indicate that a special type of chloroplast is not sufficient to establish functional kleptoplasty and intrinsic factors of the slugs also contribute to a functional photosynthesis. Here, we pave the way for further research on these specific algae consumed by LtR forms to better understand which genetically and physiological commonalities these target plastids may have. Pelletreau et al. [21] recently considered special abilities of chloroplasts from respective host algae as relevant for long-term incorporation, an option already mentioned earlier [52,53]. A factor now found to possible enhance plastid longevity is ftsH, a protein important for the repair of Photosystem II. It is plastid encoded in the food algae of the two LtR forms *Elysia chlorotica* and *Plakobranchus ocellatus*[54]. With the information of this study we are now able to specifically examine the presence of ftsH in plastids of the identified food sources of other retention forms and correlate plastid origin, photosynthetic capability and plastid longevity. Interestingly, *Thuridilla* species – as close relatives of *Plakobranchus* and *Elysia* – and which exhibit extremely short chloroplast retention [3,55], did not reveal any of these six algal species mentioned above (Additional file 1, Figure 3).

Besides plastid origin, several other factors surely influence survival of chloroplasts and render them nutritional at least in sacoglossans in one way or the other. A horizontal gene transfer as a factor was excluded not only for the sea slugs *Plakobranchus ocellatus*, *Elysia timida* and *E. chlorotica*[18,22], but also for the Foraminifera *Elphidium margaritaceum* and most likely for the dinoflagellate *Dinophysis acuminate*[56,57]. Common to all three different systems is that there is no mechanical grinding before chloroplast uptake. E.g., the sacoglossans pierce the cell wall with their teeth and then suck out the cytoplasm of the algae, which is then processed in the digestive gland. Second, the intracellular digestion of plastids seems to be stopped or regulated in a way yet to be discovered.

Händeler et al. [3] have shown in their phylogenetic analysis by using ancestral character state reconstruction, that the ancestor of the Plakobranchoidea most likely developed an unknown mechanism, which hinders direct digestion of chloroplasts. However, the genus *Costasiella* was not included in their analysis, which is now verified in comprising both, LtR and StR species ([49], [58], unpublished data). Thus, at least in *Costasiella,* this mechanism must be developed as well. Evertsen & Johnsen [59] showed that in *Elysia viridis* starch grains persist throughout degradation of plastids and are not broken up, as was the case in the NR form *Placida dendritica* (Alder & Hancock) [60]. If the slugs cannot access the photosynthetic produced starch of functional chloroplasts in the first place, the benefit, under starvation conditions, has to arise from another process. In this case the accumulated photosynthates would only become accessible for slug metabolism after the complete degradation of the chloroplast. The nutritional benefit of the chloroplasts would lie in the presence of an additional food reservoir (starch) that becomes available only after degradation or digestion of the chloroplasts. This is in contrast to former hypotheses that usually assume a continuous supply of photosynthates and therefore a continuous exchange between chloroplast and slug’s cytosol [61,62].

### General food source of Sacoglossa

Our results in combination with literature data show that sacoglossans generally feed on a high variety of algal species, although the majority prefers bryopsidophycean taxa. Jensen [4] assumed *Halimeda* as the ancestral food of Sacoglossa based on her observations on *Cylindrobulla*. We can confirm here by molecular analyses of the gut content that this species exclusively feeds on *Halimeda,* whereas all other oxynoacean species feed on *Caulerpa*, though *Lobiger viridis* seems also to at least feed additional on *Halimeda*. The position of *Cylindrobulla* within the Oxynoacea is not resolved yet, thus the ancestral food source of this clade cannot be deduced [3,63-65]. Future phylogenetic studies are needed to address these questions. All higher sacoglossan taxa switched to other food sources than *Halimeda* (especially members of the Limapontioidea), or broadened their food spectrum (many *Elysia* species).

Why some species feed on several different algal species and others specialized on just one prey species, is difficult to explain. Further information on biology has to be considered, e.g., incorporation of defensive compounds obtained via sequestration of toxic algae, or *de novo* synthesis (rendering the slug independent from algal toxins). We have to emphasize that we were not able to study all species on a broad scale by including specimens from various geographic areas or seasons. Food availability and seasonality, and/or intraspecific or interspecific competition might also force specimens from the same species to switch to other food items and thus influences the finding of certain food items in sacoglossan sea slugs. Further starvation studies on polyphagous species (see e.g. [25]) will verify if these species have a preferred food source, as was shown for *Plakobranchus ocellatus* and these investigations will certainly contribute to our understanding of polyphagous strategies [37].

Jensen [22,53] studied morphological differentiation of the radula teeth in connection with algal food. Based on her findings she suggested that certain radula teeth are correlated with the polysaccharide of cell walls of the respective food sources. However, Händeler & Wägele [23] mapped radula teeth forms on their molecular based cladogram and were not able to confirm this previously suggested correlation. We therefore compared three unusual food switches within the Sacoglossa with regard to a possible correlation of food source and radula tooth shape i) The NR form *Hermaea bifida* (Montagu) [66] feeds on the red algae *Griffithsia* with cell walls composed of cellulose. However, the radula of *H. bifida* is not different from species feeding on the green alga *Caulerpa* with cell walls composed of xylan [67]. ii) *Elysia serca*, also a NR form [68], presents a unique switch to sea grasses with cell walls composed of cellulose [28,29], but the radula is similar to species feeding on *Halimeda* with cell walls composed of xylan. iii) The NR forms *Alderia modesta* and *A. willowi,* as well as the LtR form *Elysia chlorotica,* feed on the heterokontophyte genus *Vaucheria* composed of cellulose-based cell walls. The radulae of *Alderia* species and *Elysia chlorotica* are similar to species feeding on the green alga *Codium* composed of mannan-based cell walls. Thus, we think that different radula shapes did not force or influence a host switch in these three examples, though it cannot be ruled out for others that are not examined here.

Whether functional kleptoplasty is of higher evolutionary benefit than e.g., feeding on a wide spectrum of algae may be reconsidered. A polyphagous strategy –in contrast to stenophagous strategies – would render the slugs more independent of seasonality or general availability of their host algae. LtR forms like *Costasiella ocellifera*, *Elysia chlorotica* and *Elysia timida* feed on one algal species. *Acetabularia acetabulum*, the only natural food of *E. timida*, calcifies in fall, and therefore probably forces *Elysia timida* to rely on its incorporated chloroplasts [69]. Unfortunately we have no similar information on seasonality of *Avrainvillea* and *Vaucheria*, the major food items of *C. ocellifera* and *E. chlorotica*, respectively. *Plakobranchus ocellatus* on the other side has a broad food spectrum. As Maeda et al. [37] showed in a subtle experiment, the algae show a high seasonality and the slugs combine here functional kleptoplasty and multiple food sources that certainly enhances survival.

### Usefulness of barcoding markers

**Figure 4:**
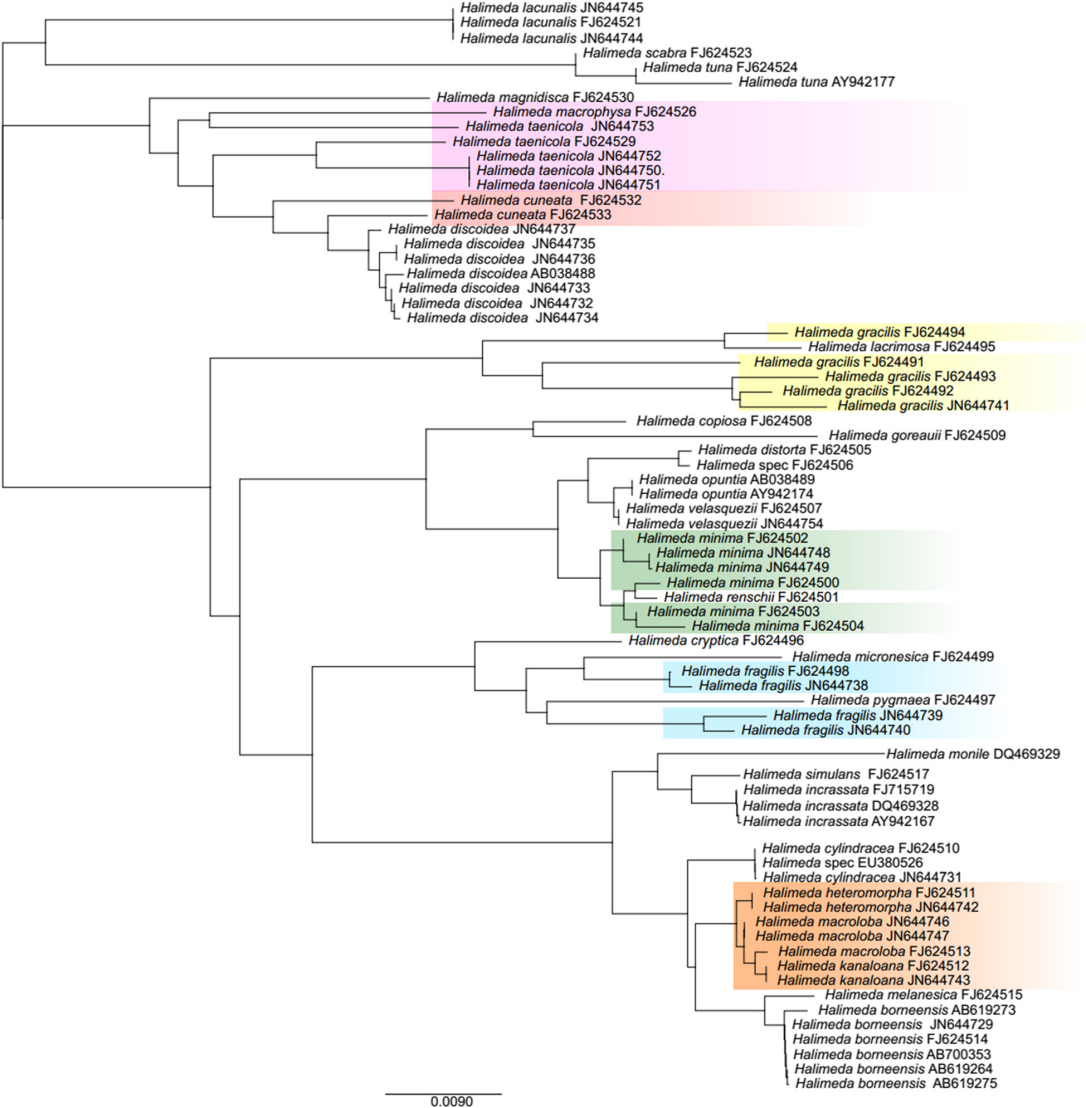
**ML tree of *rbcL *sequences of *Halimeda *species.** ML tree of randomly chosen *rbcL* sequences of *Halimeda*. Multiple haplotypes of a single species are highlighted in colored boxes.

*RbcL* and *tufA* are now state of the art markers for barcoding algal species [70] and are therefore intensively used for identification of sequestered chloroplasts in Sacoglossa [25,30,34-37]. So far it is not possible to identify the food of every sample we screened, and reasons are unknown. Christa et al. [25] showed that *rbcL* almost always revealed more haplotypes for one sacoglossan specimen than *tufA*, especially within the genus *Halimeda*. In the present study we see exactly the same pattern. We therefore compared several *rbcL* sequences of randomly chosen *Halimeda* species in order to reveal possible problems by using *rbcL* as barcoding marker for this genus (Figure 4). Our analyses show that for several *Halimeda* species various *rbcL* sequences exist. p-distances between haplotypes of the same species exceeded in some cases those of *rbcL* sequences between different species. For example, the p-distance of haplotypes of *H. cuneata* FJ624533 and FJ624532 is 2.5%, the p-distance of *Halimeda gracilis* FJ624494 and *Halimeda lacrimosa* FJ624495 1%. Assuming that there are no misidentifications (which is important for DNA-Barcoding) with regard to the sequences obtained from GenBank, this problem can lead to an overestimated number of haplotypes in cases where no reference sequences are available. With regard to our study, this might be the case for several algal sequences obtained from *Thuridilla*, which are now assigned as seven different unidentified haplotypes. It is very likely that these problems do not only occur in the taxon *Halimeda*, but also in other ulvophyceaen taxa where detailed studies are still missing. Händeler et al. [30] demonstrated for *Caulerpa* that identification and annotation using *tufA* on species level could also be problematic due to sequence similarities. Hanyuda et al. [71] reported introns in *rbcL* sequences from members of the Caulerpaceae, a problem we faced in *Caulerpa* sequences obtained from *Volvatella viridis*. These sequences cannot be aligned with the remaining *rbcL* sequences, and were therefore not included in our analyses here.

An additional problem for identifying food organisms in Sacoglossa is connected to the lack of references for both genes in the databases. Available information does not cover many of the obtained haplotypes; therefore an extension of algal taxon sampling in the future by algal specialists is absolutely necessary. Here we show that the use of two markers for plastid identification in Sacoglossa only leads to a more detailed identification of ingested plastids in some species. Yet, we suggest that, based on the higher reliability and despite possible multiple haplotypes for some genera, *rbcL* should be used preferentially. *TufA* may be added in cases where an identification of *rbcL* failed or sequence variability of *rbcL* hinders unambiguous identification. Unfortunately both primer pairs are still not applicable at the moment for Cladophoraceae [71,72]. Constructing new primers for both genes based on chloroplast genomic data probably could solve this problem as soon as a cladophoraceaen plastid genome is available. Until then it has to be kept in mind that barcoding results may cover only a reduced food range. Even with the limitations mentioned above, we consider DNA-Barcoding a more accurate and effective method than feeding observations, especially in polyphagous specimens.

## Conclusions

DNA-Barcoding of sequestered chloroplasts in Sacoglossa led to a more specific insight in food sources of sacoglossan sea slugs than by direct observation and is certainly much more efficient. *RbcL* should be used primarily because of higher amplification and sequencing success, while *tufA* should be added in analyses with ambiguous results.

Our results revealed food items for many species not investigated before and additionally confirmed literature data. We could show that food sources are highly variable in some species and morphological features such as radula shape have probably only little impact on food preferences or food switches. There seem to be certain food sources that are essential for functional kleptoplasty. LtR forms preferably consume algal species belonging to the genera *Halimeda, Caulerpa, Avrainvillea, Acetabularia* or *Vaucheria.* Kleptoplasty is only established in slugs feeding on green algae and heterokontophytes, but not in slugs feeding on rhodophytes and sea grasses. NR forms may also feed on algae that are known to be the sole food of LtR forms. Factors like physiology of food items, genetic and physiological properties of the plastids and digestion properties in slugs need to be more investigated in future studies to reveal principles of establishing functional kleptoplasty. The evolutionary benefit of kleptoplasty is still enigmatic, since a polyphagous life style may lead to more independence from specific food source’s seasonality and abundance.

## Methods

Sacoglossan specimens used in this study were identified by morphological examination using a taxonomic species file based on original literature ([73] on *Thuridilla*), as well as identification books (e.g., [74]) and the sea slug forum (http://www.seaslugforum.net). These identified specimens, covering NR, StR and LtR forms, are listed in Additional file 1 and represent those for which DNA-Barcoding of food source was successful, in order to determine the minimal food items of these species. Specimens were fixed in 96%-EtOH immediately after collection. Slug parts containing digestive glandular tissue were cut off and subsequently DNA extracted using the DNeasy® Blood & Tissue Kit (Qiagen, Germany) following manufacturer’s instructions and stored at -20°C.

### DNA amplification

Standard PCR reactions for *rbcL* and *tufA* as reported elsewhere [25,30,34-37] were performed using a touchdown protocol and ulvophycean specific primers. 2.5 μl of genomic DNA was used as template in a 20 μl final volume reaction supplied with 5.5 μl sterilized water, 2 μl Qiagen® Q-Solution, 10 μl of double concentrated QIAGEN® Multiplex PCR Master Mix and 1 μl of 5 pmol/μl concentrated primer each. PCR for amplification of *rbcL* was performed with primer pairs rbcL 1 ([36] and rbcL R, 5′-CCA WCG CAT ARA NGG TTG HGA-3′ ([25] modifid after [36]) by an initially denaturation for 15 min at 95°C, followed by 9 touch-down cycles at 94°C for 45 s, 53°C (-1°C per cycle) for 45 s, 72°C for 90 s, followed by 25 standard cycles (94°C for 45 s, 45°C for 45 s and 72°C for 90 s.) and a final extension at 72°C for 10 min. *tufA* amplification was performed with primer pair tufAF and tufAR [75] by an initially denaturation for 15 min at 95°C, followed by 9 touch-down cycles at 94°C for 45 s, 57°C (-1°C per cycle) for 45 s, 72°C for 90 s, followed by 25 standard cycles (94°C for 45 s, 48°C for 45 s and 72°C for 90 s.) and a final extension at 72°C for 10 min. PCR products were size-fractionated in a 1.5% agarose gel for 90 min at 70 V and bands according to desired gene-fragment length and subsequently gel-extracted using Machery-Nagel NucleoSpin® Extract II (Düren, Germany) kit following manufacturer’s instructions. Isolated fragments were ligated into pGEM T-easy Vector (Promega, Germany) and cloned into competent *E. coli* XL1-blue cells from Stratagene (Heidelberg, Germany). For each specimen 12 clones were sequenced by Macrogen Inc, Amsterdam.

### Sequence analysis

Sequence identity of *tufA* and *rbcL* of every clone was verified by BLAST search using Geneious (Biomatters Ltd, New Zealand, v. 6.0.3). Consensus sequences of one slug specimen were created when sequence divergence of chloroplast genes was lower than 1%, as introduced by Händeler et al. [30] (see Additional files 2, 3 and 4). All gained sequences were again verified by BLAST search and the first top 5 BLAST results of each sequence were taken to create a dataset of overall 54 sequences for *tufA* (688 bp) and 94 sequences for *rbcL* (561 bp). Both datasets were aligned with MAFFT plugin as implemented in Geneious (*v6.814b*[76]). A maximum likelihood tree for each dataset was calculated using PhyML plugin [77] implemented in Geneious with GTR + I + R as substitution model. For final identifying sequence origin we used a combination of similarity-based and tree-based DNA-barcoding approaches [78,79]: Sequences were assigned to a certain algal species by a similarity-based approach using BLAST analysis when sequence similarity was 99-100%. We included a 99% match as positive identification of an algal species, thus allowing a small amount of ambiguity nucleotides within our consensus sequences which are not identified as similarity by the BLAST search in NCBI (http://www.ncbi.nlm.nih.gov). When multiple positive identifications were obtained, sequences were assigned to the corresponding genus based on the position within the ML tree. When not able to define on species level (BLAST similarity <99%), sequences were assigned to higher taxa based on their position within monophyletic algal groups in the ML tree analysis. When sequences obtained in our study did not group within distinct algal taxa, we assigned them to the taxon level Ulvophyceae.

### Sequence analysis of *rbcL* sequences of *Halimeda*

Randomly chosen *rbcL* sequences of *Halimeda* species were downloaded from Genbank to analyze sequences variability. Overall 72 sequences were used, representing 32 species. Dataset was aligned with MAFFT plugin as implemented in Geneious (*v6.814b*[76]). A maximum likelihood tree for the dataset was calculated using PhyML plugin [77] implemented in Geneious with GTR + I + R as substitution model. P-distances of sequences were calculated using HyPhy (v 2.1). On the basis of these p-distances the sequence variability of haplotypes of the same algal species were then analyzed.

### Comparison of literature data and new barcoding data

DNA-Barcoding results of the present study were combined with results taken from literature to compare food sources of different retention forms (LtR, StR, NR). Literature data on food sources and retention form were taken from [3,4,18,25,30,34-37,80-84] (Additional files 1 and 5). The retention ability of the majority of the species investigated here was measured by means of a PAM. Therefore, we used the classification scheme of Händeler et al. [3]. Results of Clark et al. [68] based on ^14^C methods were re-assigned, when new data were available. For example, *Mourgona germaineae* Marcus, 1970 is classified as StR form by Clark et al. [68], however PAM measurements revealed no fluorescence at all (unpublished data). We therefore set ^14^C obtained by values similar to *M. germaineae* as “no retention”, e.g. *Caliphylla mediterranea* Costa [85], *Hermaea cruciata*, *Placida kingstoni* Thompson [86] and *Elysia serca*. Most recently Schmitt et al. [87] could show that *E. timida* can be artificially raised on *Acetabularia peniculus*, an algal species that naturally does not co-occur with the slugs. Similar retention of plastids was observed as from the natural food A.

## Competing interests

The authors declare that they have no competing interests.

## Authors’ contributions

HW, KH and GC conceived and designed the experiment. GC, TS and KH performed the experiments. HW, GC and KH analyzed the data. GC, TS, GK and HW wrote the paper. All authors read and approved the final manuscript.

## Supplementary Material

Additional file 1 **Origin of specimen and identified food sources with comparison of literature data.** In **Table S1** we provide a list of Sacoglossa specimens and species analyzed with regard to food items. **Table S2** displays the identified food sources in Sacoglossa specimens by analyzing the chloroplast markers *tufA* and *rbcL*. The number of haplotypes per gene obtained from each slug specimen is listed in the last two columns. * *tufA* sequences of *Thuridilla hopei* (809), Vérany were published previously [18]. **Table S3** shows a comparison of chloroplast origin by feeding observations (4^th^ column) and identified by DNA-Barcoding (this study and literature data – last column). When not specified specifically, information on retention-form is taken from Händeler et al. [3]. Food sources identified by using *tufA* are indicated with ^1^, by *rbcL* with ^2^ in the last column. Literature data on food sources base on the review of Händeler & Wägele [29] or is indicated otherwise. Click here for file

Additional file 2 Sequence affinity based on BLAST search. Click here for file

Additional file 3 **Alignment of *tufA *sequences used for consensus sequences.** Click here for file

Additional file 4 **Alignment of *rbcL *sequences used for consensus sequences.** Click here for file

Additional file 5 Classification on retention form in Sacoglossa. Click here for file
