# Supplementary material for: Identification of sequestered chloroplasts in photosynthetic and non-photosynthetic sacoglossan sea slugs (Mollusca, Gastropoda)
Source: Front Zool. 2014 Feb 21;11:15. doi: 10.1186/1742-9994-11-15 (PMC3941943; doi:10.1186/1742-9994-11-15)
Supplement: Additional file 1 — Origin of specimen and identified food sources with comparison of literature data. In Table S1 we provide a list of Sacoglossa specimens and species analyzed with regard to food items. Table S2 displays the identified food sources in Sacoglossa specimens by analyzing the chloroplast markers tufA and rbcL. The number of haplotypes per gene obtained from each slug specimen is listed in the last two columns. * tufA sequences of Thuridilla hopei (809), Vérany were published previously [18]. Table S3 shows a comparison of chloroplast origin by feeding observations (4th column) and identified by DNA-Barcoding (this study and literature data – last column). When not specified specifically, information on retention-form is taken from Händeler et al. [3]. Food sources identified by using tufA are indicated with 1, by rbcL with 2 in the last column. Literature data on food sources base on the review of Händeler & Wägele [29] or is indicated otherwise. [file 1742-9994-11-15-S1.docx]

**Table S1 – Origin of Sacoglossa species**

|  | Species name | Specimen (internal number) | Origin | Accession Numbers | |
| --- | --- | --- | --- | --- | --- |
|  |  |  |  | *tufA* | *rbcL* |
| **Oxynoacea** | *Cylindrobulla* sp. | 847 | USA, Guam: Cocos Lagoon | KC479636 | KC479619 |
|  | *Lobiger viridis* | S111 | Australia: Lizard Island, Casuarina Beach | KC479642 | KC479604 |
|  | *Volvatella viridis* | 858 | Australia: Lizard Island, Casuarina Beach | KJ002536 | - |
| **Limapontioidae** | *Costasiella kuroshimae* | S190 | Australia: Lizard Island, Casuarina Beach | KC479649 | KC479593 |
|  | *Costasiella kuroshimae* | S196 | Australia: Lizard Island, Casuarina Beach | KC479650 | KC479591 |
|  | *Costasiella* sp. 1 | 864 | USA, Guam: Cocos Lagoon | KC479637 | KC479608  KC479609  KC479610  KC479611 |
|  | *Costasiella* sp. 2 | 863 | USA, Guam, Cocos Lagoon |  | KC479612  KC479613 |
|  | *Cyerce nigra* | 860 | USA, Guam: Cocos Lagoon |  | KC479614  KC479615  KC479616  KC479617  KC479618 |
|  | *Ercolania viridis* | S150 | France: Banyuls-sur-Mer | KC479648 |  |
| **Plakobranchoidae** | *Elysia amakusana* | 703 | Australia: Lizard Island |  | KC479631 |
|  | *Elysia asbecki* | S280 | Australia: Lizard Island, Horseshoe Reef | KC479651  KC479652 | KC479587  KC479588  KC479589  KC479590 |
|  | *Elysia benettae* | S107 | Australia: Lizard Island, Horseshoe Reef | KC479641 | KC479605  KC479606 |
|  | *Elysia obtusa* | 778 | Samoa, Savii: Savaii Island: Vaisala Lagoon |  | KC479583  KC479584  KC479585  KC479586 |
|  | *Elysia ornata* | S123 | Australia: Lizard Island, Vicky Reef |  | KC479594  KC479595  KC479596 |
|  | *Elysia* sp. | 865 | USA, Guam: Asan | KC479638 |  |
|  | *Elysia* sp. | 871 | USA, Guam: Apra Harbour | KC479639  KC479640 | KC479607 |
|  | *Elysia* sp. | 841 | France: Banyuls-sur-Mer |  | KC479623  KC479624 |
|  | *Elysia timida* | 743 | Spain: Plaja Caials | KC479633 | KC479582 |
|  | *Elysia translucens* | 845 | France: Banyuls-sur-Mer |  | KC479622 |
|  | *Thuridilla albopustulosa* | 782 | Indonesia, Sulawesi: S-Siladen |  | KC479625  KC479626 |
|  | *Thuridilla bayeri* | 725 | Indonesia: NW-Sulawesi |  | KC479628  KC479629  KC479630 |
|  | *Thuridilla bayeri* | S192 | USA, Guam: Cocos Lagoon |  | KC479592 |
|  | *Thuridilla carlsoni* | S116 | Australia: Lizard Island, Loomis Reef | KC479643  KC479644 |  |
|  | *Thuridilla gracilis* | S117 | Australia: Lizard Island, Casuarina Beach | KC479645 | KC479601  KC479602  KC479603 |
|  | *Thuridilla gracilis* | S118 | Australia: Lizard Island, Casuarina Beach | KC479646  KC479653  KC479654 | KC479600 |
|  | *Thuridilla gracilis* | S122 | Australia: Lizard Island, Casuarina Beach |  | KC479597  KC479598  KC479599 |
|  | *Thuridilla hoffae* | 777 | Samoa, Savii;: Savaii Island: Vaisala lagoon |  | KC479627 |
|  | *Thuridilla hopei* | 809 | Italy: Isola del Giglio, Cala del Corvo |  | KC479632 |
|  | *Thuridilla kathae* | S120 | Australia: Lizard Island, Casuarina Beach | KC479647 |  |
|  | *Thuridilla livida* | 846 | USA, Guam: Bile Bay | KC479634  KC479635 | KC479620  KC479621 |

**Table S2 – Food sources of Sacoglossa identified by DNA-Barcoding**

|  |  | Food source identified | | Number of haplotypes | |
| --- | --- | --- | --- | --- | --- |
|  |  | *tufA* | *rbcL* | *tufA* | *rbcL* |
| **Oxynoacea** | *Cylindrobulla* sp. (847) | *Halimeda macroloba* | *Halimeda* sp. 2 | 1 | 1 |
|  | *Lobiger viridis* (S111) | *Caulerpa serrulata* | *Halimeda* sp. 1 | 1 | 1 |
|  | *Volvatella viridis* (858) | *Caulerpa serrulata* | *Caulerpa* sp. | 1 | 1 |
| **Limapontioidea** | *Costasiella kuroshimae* (S190) | *Avrainvillea* sp. | *Avrainvillea* sp. 1 | 1 | 1 |
|  | *Costasiella kuroshimae* (S196) | *Avrainvillea* sp. | *Avrainvillea* sp. 1 | 1 | 1 |
|  | *Costasiella* sp. (863) | - | *Tydemania* sp.  Rhipiliacea sp. 1 | - | 2 |
|  | *Costasiella* sp. (864) | *Tydemania* sp. 1 | *Avrainvillea* sp.1  *Pseudochlorodesmis* sp. 2  Rhipiliacea sp. 2 | 1 | 3 |
|  | *Cyerce nigra* (860) | - | *Bryopsis* sp. 1  *Avrainvillea* sp. 1  *Ulva* sp. 1  *Pseudochlorodesmis* sp. 2 *Pseudochlorodesmis* sp. 3 | - | 5 |
|  | *Ercolania viridis* (S150) | *Ulva intestinalis* | - | 1 | - |
| **Plakobranchoidea** | *Elysia amakusana* (703) | - | *Halimeda* sp. 2 | - | 1 |
|  | *Elysia asbecki* (S280) | Ulvophyceae sp. 3 *Rhipiliaceae* sp. 2 | Rhipiliacea sp. 2  *Rhipiliopsis* sp. 1  *Rhipiliopsis* sp. 2  *Caulerpella* sp. | 2 | 4 |
|  | *Elysia benettae* (S107) | *Halimeda* sp. 1 | *Halimeda* sp.1  Ulvophyceae sp. 9 | 1 | 2 |
|  | *Elysia obtusa* (778) | - | *Polyphysa amgigua*  *Halimeda* sp. 1  *Tydemania expeditionis*  *Bryopsis* sp. 2 |  | 4 |
|  | *Elysia ornata* (S123) | - | *Bryopsis* sp. 4  *Bryopsis* sp. 5  *Bryopsis* sp. 6 | - | 3 |
|  | *Elysia* sp*.* (865) | *Tydemania expeditionis* | - | 1 | - |
|  | *Elysia* sp. (871) | *Halimeda* sp.1  *Halimeda* sp.2 | *Halimeda* sp. 1 | 2 | 1 |
|  | *Elysia* sp. (841) | - | *Pseudochlorodesmis* sp. 3  *Pseudochlorodesmis furcellata* | - | 2 |
|  | *Elysia timida* (743) | *Acetabularia acetabulum* | *Acetabularia acetabulum* | 1 | 1 |
|  | *Elysia translucens* (845) | - | *Bryopsis* sp. 3 | - | 1 |
|  | *Thuridilla albopustulosa* (782) | - | Ulvophyceae sp. 5 | - | 1 |
|  | *Thuridilla bayeri* (725) | - | Ulvophyceae sp. 5  Ulvophyceae sp. 6  Ulvophyceae sp. 7 | - | 3 |
|  | *Thuridilla bayeri* (S192) | - | *Pseudochlorodesmis* sp. 2 | - | 1 |
|  | *Thuridilla carlsoni* (S116) | *Rhipiliaceae* sp. 1 *Rhipiliaceae* sp. 2 | - | 2 | - |
|  | *Thuridilla gracilis* (S117) | Ulvophyceae sp. 2 | *Pseudochlorodesmis* sp. 1  *Pseudochlorodesmis* sp. 2 Ulvophyceae sp. 8 | 1 | 3 |
|  | *Thuridilla gracilis* (S118) | Ulvophyceae sp. 2  *Udotaceae* sp.  *Codium* sp. | *Pseudochlorodesmis* sp. 2 | 3 | 1 |
|  | *Thuridilla gracilis* (S122) | - | *Pseudochlorodesmi*s sp. 2  *Halimeda* sp. 1  Ulvophyceae sp. 9 | - | 3 |
|  | *Thuridilla hoffae* (777) | - | Ulvophyceae sp. 2 | - | 1 |
|  | *Thuridilla hopei* (809) | Uncultured Ulvophyceae* | Ulvophyceae sp. 4 | * | 1 |
|  | *Thuridilla kathae* (S120) | *Rhipiliaceae* sp. 1 | - | 1 | - |
|  | *Thuridilla livida* (846) | Ulvophyceae sp. 2 Ulvophyceae sp. 3 | *Pseudochlorodesmis* sp. 2 Ulvophyceae sp. 3 | 2 | 2 |

**Table S3 – Chloroplast origin in Sacoglossa**

|  |  | Retention-Form | Food identified via feeding experiments or observations | Food identified via DNA-Barcoding |
| --- | --- | --- | --- | --- |
| **Oxynoacea** | *Cylindrobulla* sp. | No | ? | *Halimeda macroloba^1,2^* |
|  | *Lobiger viridis* | No | ? | *Caulerpa serrulata^1^* |
|  | *Volvatella viridis* | No | *Caulerpa* [4] | *Caulerpa racemosa^1^*  *Caulerpa serrulata^1,2^*  [30] |
| **Limapontioidea** | *Costasiella kuroshimae* | No | *Avrainvillea*  *Codium* | *Avrainvillea* sp. *^1,2^* |
|  | *Costasiella* sp. (863) | No | ? | Rhipiliacea sp.*^2^*  *Tydemania expeditionis^2^* |
|  | *Costasiella* sp.  (864) | No | ? | *Avrainvillea* sp.*^2^*  *Pseudochlorodesmis* sp.*^2^*  Rhipiliacea sp.*^2^*  *Tydemania expeditionis^1^* |
|  | *Cyerce nigra* | No | *Chlorodesmis comosa* [88] | *Avrainvillea* sp.*^2^*  *Bryopsis* sp.*^2^*  *Pseudochlorodesmis* spp*^1^*  *Ulva* sp.*^2^* |
|  | *Ercolania viridis* | No | *Chaetomorpha* spp.  *Cladophora* spp. | *Ulva intestinalis^1^* |
| **Plakobranchoidea** | *Bosellia mimetica* | Short | *Halimeda*  *Udotea* | *Halimeda tuna^1^* [30] |
|  | *Elysia amakusana* | Short | ? | *Halimeda macroloba^2^* |
|  | *Elysia asbecki* | Short | ? | *Caulerpella ambigua^2^*  *Rhipiliaceae* spp.*^1,2^*  Ulvophyceae sp. *^1^* |
|  | *Elysia benettea* | Short | *Chlorodesmis* spp | *Halimeda minima^1,2^*  Ulvophyceae sp. *^2^* |
|  | *Elysia clarki* | Long | ? | *Halimeda incrassata^2^*  *Halimeda monile^2^* [35]  *Penicillus capitatus^2^*  *Penicillus lamourouxii^2^* |
|  | *Elysia crispata* | Long | *Batophora*  *Bryopsis*  *Caulerpa*  *Halimeda*  *Penicillus* | *Chlorodesmis* sp.*^2^* [36]  *Halimeda* spp. *^2^*  *Halimeda incrassata^2^*  *Penicillus* spp.*^2^*  *Penicillus capitatus^2^* |
|  | *Elysia macnaei* | Short | *Halimeda cuneata*  *Halimeda macroloba* | *Halimeda macrophysa^1^* [30] |
|  | *Elysia obtusa* | Short | ? | *Bryopsis* sp.*^2^*  *Halimeda minima^2^*  *Polyphysa ambigua^2^*  *Tydemania expeditionis^2^* |
|  | *Elysia ornata* | Short | *Bryopsis* | *Bryopsis* spp.*^2^* |
|  | *Elysia pusilla* | Short | *Halimeda* spp. | *Halimeda opuntia^1^* [30] |
|  | *Elysia* sp. (865) | Short | ? | *Tydemania expeditionis^1^* |
|  | *Elysia* sp. (871) | Short | ? | *Halimeda macroloba^2^*  *Halimeda minima^1,2^* |
|  | *Elysia* sp*.* (841) | Short | ? | *Pseudochlorodesmis* sp. 3*^2^*  *Pseudochlorodesmis furcellata^2^* |
|  | *Elysia timida* | Long | *Acaetabularia acetabulum*  *Cladophora* spp.  *Codium* spp. | *Acetabularia acetabulum^1,2^* |
|  | *Elysia tomentosa* | Short |  | *Caulerpa racemosa^1^* [30] |
|  | *Elysia translucens* | Short | *Udotea petiolata* | *Bryopsis* sp.*^2^* |
|  | *Elysia viridis* | Short | *Bryopsis* spp.  *Chaetomorpha*  *Cladophora*  *Codium* spp. | *Bryopsis plumosa^1^* [30] |
|  | *Plakobranchus ocellatus* | Long | ? | *Acetabularia acetabulum^1,2^*  *Caulerpa* spp.*^1,2^*  *Caulerpella* spp.*^1,2^*  *Codium* sp. *^1,2^*  *Halimeda* spp*.^1,2^*  *Pseudochlorodesmis* spp.*^1,2^*  *Proposis* spp.*^1,2^*  *Rhipidosiphon* spp.*^1,2^*  *Udotea* spp.*^1,2^*  [18, 25, 37] |
|  | *Thuridilla albopustulosa* | Short | ? | *Ulvopyceae* sp. *^2^* |
|  | *Thuridilla bayeri* | Short | ? | *Pseudochlorodesmis* sp.*^2^*  Ulvopyceae spp.*^2^* |
|  | *Thuridilla carlsoni* | Short | ? | *Rhipiliaceae* spp.*^1^* |
|  | *Thurdilla gracilis* | Short | ? | *Codium* sp.*^1^*  *Halimeda minima^2^*  *Pseudochlorodesmis* spp*^2^*  *Udotea* sp.*^1^*  Ulvophyceae spp.*^1,2^* |
|  | *Thuridilla hoffae* | Short | ? | *Ulvophyceae* sp.*^2^* |
|  | *Thuridilla hopei* | Short | *Cladophora vagabunda*  *Derbesia tenuissima* | *Ulvopyceae* sp.*^1,2^*  [18] |
|  | *Thuridilla kathae* | Short | ? | *Rhipiliaceae* sp.*^1^* |
|  | *Thuridilla livida* | Short | ? | *Pseudochlorodesmis* sp.*^2^*  *Ulvopyceae* spp.*^1,2^* |
