# Supplementary material for: Identification of sequestered chloroplasts in photosynthetic and non-photosynthetic sacoglossan sea slugs (Mollusca, Gastropoda)
Source: Front Zool. 2014 Feb 21;11:15. doi: 10.1186/1742-9994-11-15 (PMC3941943; doi:10.1186/1742-9994-11-15)
Supplement: Additional file 5 — Classification on retention form in Sacoglossa. [file 1742-9994-11-15-S5.docx]

Additional file 5 Classification of the retention form of Sacoglossa based on Chlorophyll fluorescence measurements (PAM), CO2 fixation experiments (^14^C) or oxygen evolution (O_2_). The classification in non-retention (NR, grey), short-term-retention (StR, blue) and long-term-retention (LtR, green) is based on Händeler *et al.* [1]. Previous classification of Clark *et al.* [2] were re-defined based on new findings (see Material sections for details). When no data is published the classification in retention form is done by estimation (indicated by * at the retention form).

| Taxon | Species | Retention form | Method | Reference |
| --- | --- | --- | --- | --- |
|  |  |  |  |  |
| Oxynoacea | *Cylindrobulla sp*. | **NR** | PAM | [3] |
|  | *Cylindrobulla beauii* | **NR** | PAM | Unpublished data |
|  | *Oxynoe viridis* | **NR** | PAM | [1] |
|  | *Oxynoe antillarum* | **NR** | PAM | [1] |
|  | *Julia exquisita* | **NR** | PAM | [1] |
|  | *Lobiger viridis* | **NR** | PAM | [1] |
|  | *Volvatella viridis* | **NR** | PAM | [1] |
|  | *Tamanovalva limax* | **NR*** |  |  |
|  |  |  |  |  |
| Limapontioidea | *Aplysiopsis enteromorphae* | **NR*** |  |  |
|  | *Hermeae bifida* | **NR** | ^14^C | [4] |
|  | *Hermaea cruciate* | **NR** | ^14^C | [2] |
|  | *Costasiella* sp. 863 | **NR** | PAM | [3] |
|  | *Costasiella* sp. 864 | **StR** | PAM | [3] |
|  | *Costasiella nonatoi* | **NR** | PAM | Christa et al. (submitted) |
|  | *Costasiella ocellifera* | **LTR** | PAM | Christa et al. (submitted) |
|  | *Polybranchia orientalis* | **NR*** |  |  |
|  | *Mourgona germaineae* | **NR** | ^14^C | [2] |
|  | *Cyerce antillensis* | **NR** | PAM | Unpublished data |
|  | *Cyerce nigra* | **NR** | PAM | [3] |
|  | *Cyerce nigricans* | **NR** | PAM | [1] |
|  | *Caliphylla mediterranea* | **NR** | ^14^C | [2] |
|  | *Placida dendritica* | **NR** | PAM | [1] |
|  | *Placida kingstoni* | **NR** | ^14^C | [2] |
|  | *Stilligher berghi* | **NR** | PAM | [5] |
|  | *Limapontia capitata* | **NR** | ^14^C | [6] |
|  | *Limapontia depressa* | **NR** | ^14^C | [6] |
|  | *Ercolania annelyleorum* | **NR** | PAM | [7] |
|  | *Ercolania boodle* | **NR** | PAM | [5] |
|  | *Ercolania fuscata* | **NR** | PAM | Unpublished data |
|  | *Ercolania kencolesi* | **NR** | PAM | [1] |
|  | *Ercolania viridis* | **NR** | PAM | [3] |
|  | *Calliopaea oophaga* | **NR** |  |  |
|  | *Alderia modesta* | **NR** | ^14^C | [6] |
|  | *Alderia willowi* | **NR** | PAM | Unpublished data |
| Plakobranchoidea | *Bosellia mimetica* | **StR** | PAM | [1] |
|  | *Elysia amakusana* | **StR*** |  |  |
|  | *Elysia artroviridis* | **StR** | PAM | [5] |
|  | *Elysia asbecki* | **StR** | PAM | [7] |
|  | *Elysia benettae* | **StR** | PAM | [1] |
|  | *Elyisa chlorotica* | **LtR** | O_2_ | [8] |
|  | *Elysia clarki* | **LtR** | PAM | [9] |
|  | *Elysia crispata* | **LtR** | PAM | [1] |
|  | *Elysia cornigera* | **StR** | PAM | [1] |
|  | *Elysia macnaei* | **StR*** |  |  |
|  | *Elysia nicrocapitata* | **StR** | PAM | [10] |
|  | *Elysia obtusa* | **StR** |  |  |
|  | *Elysia ornata* | **StR** | PAM | [1] |
|  | *Elysia patina* | **StR** | PAM | Unpublished data |
|  | *Elysia pusilla* | **StR** | PAM | [1] |
|  | *Elysia serca* | **NR** | ^14^C | [2] |
|  | *Elysia* sp. 841 | **StR** | PAM | [3] |
|  | *Elysia* sp. 865 | **StR** | PAM | [3] |
|  | *Elysia* sp. 871 | **StR** | PAM | [3] |
|  | *Elysia subornata* | **NR** | PAM | [1] |
|  | *Elysia timida* | **LtR** | PAM | [1] |
|  | *Elysia tomentosa* | **StR** | PAM | [1] |
|  | *Elysia trisinuata* | **StR** | PAM | [11] |
|  | *Elysia tuca* | **StR** | PAM | Unpublished data |
|  | *Elysia viridis* | **StR** | PAM | [12] |
|  | *Plakobranchus ocellatus* | **LtR** | PAM | [1] |
|  | *Thuridilla albopustulosa* | **StR*** |  |  |
|  | *Thuridilla bayeri* | **StR** | PAM | [3] |
|  | *Thuridilla carlsoni* | **StR** | PAM | [1] |
|  | *Thuridilla gracilis* | **StR** | PAM | [1] |
|  | *Thuridilla hoffae* | **StR*** |  |  |
|  | *Thuridilla hopei* | **StR** | PAM | [1] |
|  | *Thuridilla kathae* | **StR** | PAM | [1] |
|  | *Thuridilla livida* | **StR** | PAM | [3] |

**References**

1. Händeler K, Grzymbowski YP, Krug PJ, Wägele H: **Functional chloroplasts in metazoan cells - a unique evolutionary strategy in animal life**. *Front Zool* 2009, **6**:28.

2. Clark KB, Jensen KR, Stirts HM: **Survey for functional kleptoplasty among west Atlantic Ascoglossa (= Sacoglossa) (Mollusca : Opisthobranchia)**. *The Veliger* 1990, **33**:339–345.

3. Händeler K: **Evolution of Sacoglossa (Opisthobranchia) with emphasis on their food (Ulvophyceae) and the ability to incorporate kleptoplasts**. 2011:1–134.

4. Kremer BP, Schmitz K: **Aspects of 14CO2-fixation by endosymbiotic rhodoplasts in the marine opisthobranchiate Hermaea bifida**. *Mar Biol* 1976, **34**:313–316.

5. Klochkova TA, Han J-W, Kim J-H, Kim K-Y, Kim G-H: **Feeding specificity and photosynthetic activity of Korean sacoglossan mollusks**. *Algae* 2010, **25**:217–227.

6. Hinde R, Smith DC: **“Chloroplast symbiosis” and the extent to which it occurs in Sacoglossa (Gastropoda: Mollusca)**. *Biol J Linn Soc* 1974, **6**:349–356.

7. Wägele H, Stemmer K, Burghardt I, Haendeler K: **Two new sacoglossan sea slug species (Opisthobranchia, Gastropoda): *Ercolania annelyleorum* sp. nov.(Limapontioidea) and *Elysia asbecki* sp. nov.(Plakobranchoidea), with notes on anatomy, histology and biology**. *Zootaxa* 2010, **2676**:1–28.

8. Rumpho ME, Summer EJ, Manhart JR: **Solar-powered sea slugs. Mollusc/algal chloroplast symbiosis.** *Plant Physiology* 2000, **123**:29–38.

9. Middlebrooks ML, Pierce SK, Bell SS: **Foraging Behavior under Starvation Conditions Is Altered via Photosynthesis by the Marine Gastropod, Elysia clarki**. *PLoS ONE* 2011, **6**:e22162.

10. Klochkova TA, Han J-W, Chah K-H, Kim RW, Kim J-H, Kim K-Y, Kim G-H: **Morphology, molecular phylogeny and photosynthetic activity of the sacoglossan mollusc, *Elysia nigrocapitata*, from Korea**. *Mar Biol* 2012, **160**:155–168.

11. Yamamoto YY, Yusa Y, Yamamoto S, Hirano Y, Hirano Y, Motomura T, Tanemura T, Obokata J: **Identification of photosynthetic sacoglossans from Japan**. *Endocytobiosis Cell Res* 2009, **19**:112–119.

12. Evertsen J, Johnsen G: **In vivo and in vitro differences in chloroplast functionality in the two north Atlantic sacoglossans (Gastropoda, Opisthobranchia) *Placida dendritica* and *Elysia viridis***. *Mar Biol* 2009, **156**:847–859.
